# Edematous wall thickening of the gallbladder induced by hyperthyroidism: A case report

**DOI:** 10.1097/MD.0000000000028720

**Published:** 2022-01-28

**Authors:** Katsumasa Koyama, Takatoshi Anno, Fumiko Kawasaki, Ken Nishino, Hirofumi Kawamoto, Hideaki Kaneto, Koichi Tomoda

**Affiliations:** a Department of General Internal Medicine 1, Kawasaki Medical School, Okayama, Japan; b Department of General Internal Medicine 2, Kawasaki Medical School, Okayama, Japan; c Department of Diabetes, Endocrinology and Metabolism, Kawasaki Medical School, Kurashiki, Japan.

**Keywords:** Basedow disease, edematous wall thickening of the gallbladder, fluid retention, hyperthyroidism

## Abstract

**Rationale:**

Hyperthyroidism, such as Basedow disease, causes fluid retention, although the common cause is volume overload due to congestive heart failure. In addition, hyperthyroidism and Basedow disease are known to cause pulmonary hypertension. Edematous thickening of the gallbladder wall is caused by venous blood congestion. The feature of edematous wall thickening of the gallbladder on abdominal computed tomography (CT) is subserosal edema and is often accompanied by a periportal collar sign.

**Patient concerns:**

A 30-year-old woman was referred to our hospital because of liver dysfunction, edematous gallbladder wall thickening, and fluid retention. In addition, the patient developed hyperthyroidism and heart failure. Enhanced abdominal CT revealed edematous wall thickening of the gallbladder and a periportal collar sign.

**Diagnosis:**

We suspected that fluid retention and congestion were caused by hyperthyroidism and Basedow disease.

**Interventions:**

On admission, we started thiamazole therapy for Basedow disease, and her thyroid hormone levels normalized.

**Outcomes:**

Abdominal CT revealed disappearance of edematous wall thickening of the gallbladder, which was likely associated with an improvement in thyroid function. The patient was discharged 10 days after admission.

**Lessons:**

We encountered a case of hyperthyroidism and Basedow disease accompanied by edematous wall thickening of the gallbladder and various fluid retentions as the first symptoms. Such edematous wall thickening of the gallbladder and various fluid retentions were reduced, together with the improvement of hyperthyroidism.

## 1 Introduction

Fluid retention is sometimes observed in patients with thyroid disease, especially with hypothyroidism.^[1]^ Under hypothyroid conditions, edema is caused by a reduction in enzymatic activity, resulting in a buildup of mucopolysaccharides, which is called nonpitting edema. On the other hand, hyperthyroidism, such as Basedow disease, rarely causes fluid retention, although the common cause is volume overload due to congestive heart failure.^[2]^ In addition, it is known that hyperthyroidism and Basedow disease sometimes cause pulmonary hypertension.^[3]^ Moreover, hypoproteinemia and iron deficiency anemia can be additional factors for fluid retention under hyperthyroid conditions.

Edematous wall thickening of the gallbladder is caused by venous blood congestion, which is associated with heart failure, hypoproteinemia, acute hepatitis, and liver cirrhosis.^[4]^ The feature of edematous wall thickening of the gallbladder on abdominal computed tomography (CT) is subserosal edema because the connective tissue around the gallbladder is thin and dense. Edematous wall thickening of the gallbladder differs from infectious wall thickening of the gallbladder, which is sometimes caused by acute cholecystitis. In addition, edematous wall thickening of the gallbladder is often accompanied by a periportal collar sign, which likely represents periportal edema.^[5]^

In this report, we show a subject with edematous wall thickening of the gallbladder and various fluid retention induced by hyperthyroidism and such edematous wall thickening of the gallbladder was the first symptom for diagnosis of Basedow disease. Her edematous wall thickening of the gallbladder and various fluid retentions were improved, which were likely associated with decreased thyroid hormone levels.

## 2 Case presentation

**Figure 1:**
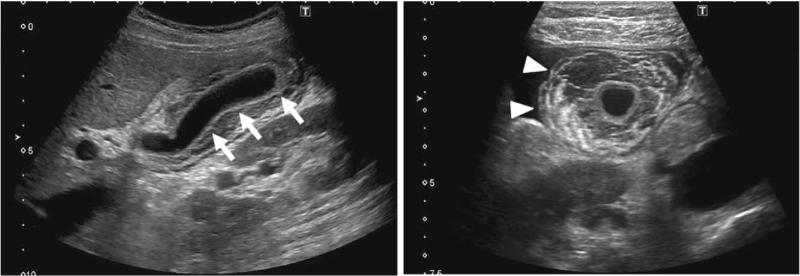
Abdominal ultrasonography. Edematous wall thickening of the gallbladder is detected (12.0 mm) (white arrow), and the inner cavity is not enlarged, although the gallbladder is enlarged in diameter (44.0 mm). In addition, edematous wall thickening of the gallbladder was observed, accompanied by subserosal edema (white arrowhead).

**Figure 2:**
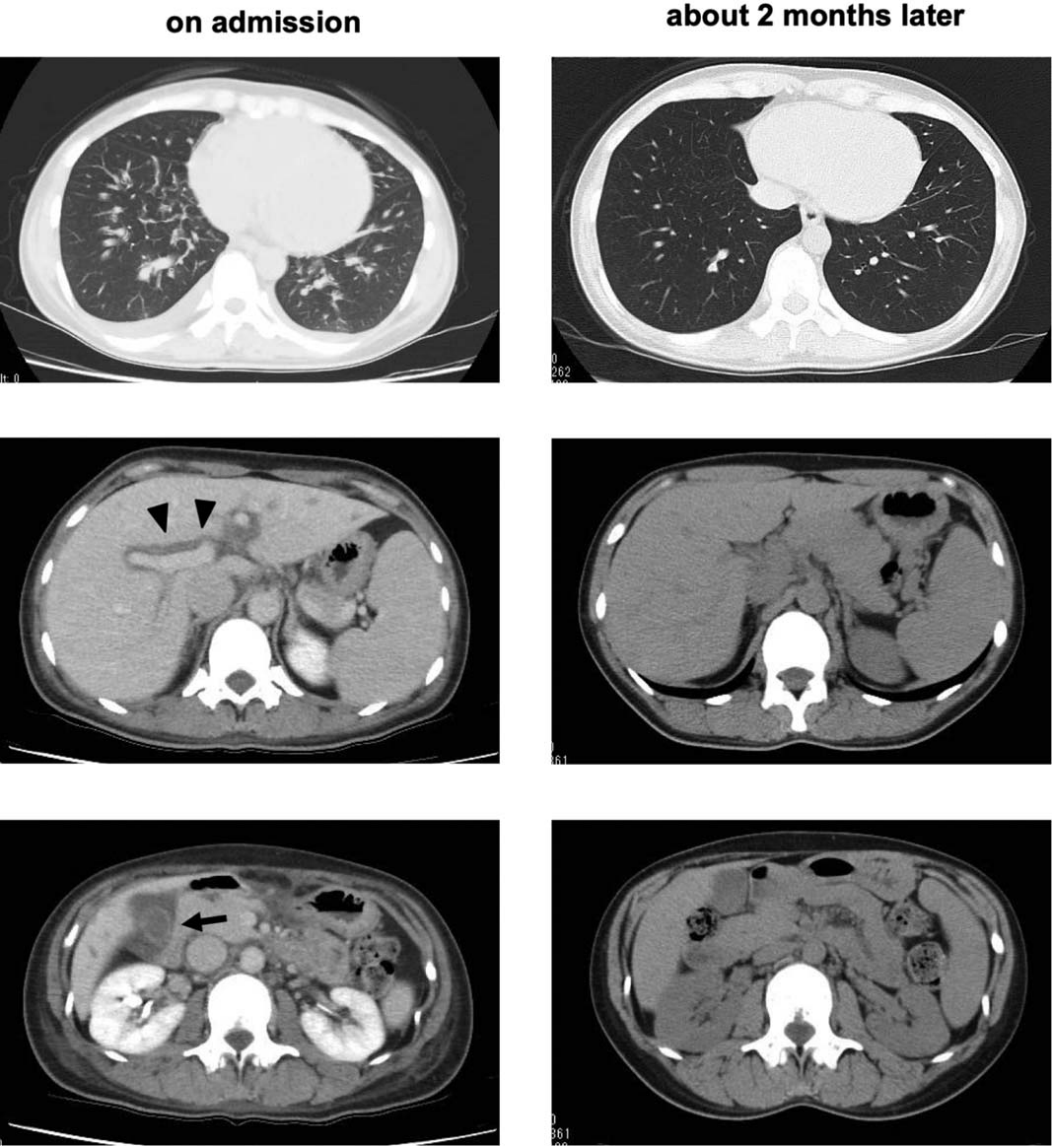
Complete disappearance of fluid retention and congestion. Enhanced chest and abdominal computed tomography (CT): Left panel, enhanced chest and abdominal CT on admission; right panel, chest and abdominal CT about 2 mo later. Enhanced chest and abdominal CT on admission revealed pleural effusion, ascites and splenomegaly. In addition, edematous wall thickening of the gallbladder (black arrow) and periportal collar sign (black arrow head) in enhanced abdominal CT. On the other hand, chest and abdominal CT about 2 mo later revealed the disappearance of edematous wall thickening of the gallbladder which was likely associated with the improvement of thyroid function.

A 30-year-old Japanese woman was referred to our hospital because of liver dysfunction, edematous wall thickening of the gallbladder, and fluid retention on an abdominal CT. She had no remarkable or family history. She was a nonsmoker and did not drink alcohol. The patient height and body weight were 164.2 cm and 48.8 kg, respectively. Her vital signs were as follows: temperature, 36.0°C; blood pressure, 130/60 mm Hg; heart rate, 60 bpm; and oxygen saturation, 98%. Table 1 shows the laboratory data on admission. She had iron deficiency anemia (red blood cells, 397 × 10^4^/μL; Hemoglobin, 9.8 g/dL; iron, 17 μg/dL; ferritin, 7 ng/mL). Renal function was within the normal range, but liver dysfunction was observed as follows: aspartate aminotransferase, 109 U/L; alanine transaminase, 178 U/L; alkaline phosphatase, 115 U/L; γ-glutamyl transpeptidase, 33 U/L; and lactate dehydrogenase, 166 U/L. Moreover, the brain natriuretic peptide (BNP) level, a marker of heart failure, was markedly elevated to 565.9 pg/mL. Chest radiography revealed a Cardio-Thoracic Ratio of 50.72%. Echocardiography revealed an increase in the inferior vena cava diameter (22.8 mm) and pulmonary hypertension. The ejection fraction was 67%. Abdominal ultrasonography revealed edematous wall thickening of the gallbladder and subserosal edema (Fig. 1). In addition, enhanced chest and abdominal CT revealed edematous wall thickening of the gallbladder and a periportal collar sign, which likely represented periportal edema, together with pleural effusion, ascites, and splenomegaly (Fig. 2, left panel). Since her BNP level was markedly elevated, we thought that her main pathology was fluid retention and congestion accompanied by heart failure and pulmonary hypertension. Hepatitis virus antibodies showed a pattern of prior infection. The thyroid-associated data were as follows: thyroid-stimulating hormone, <0.010 μU/mL; free triiodothyronine, 8.14 pg/mL; free thyroxine, 2.02 ng/dL; thyroid-stimulating hormone receptor antibody, 8.7 U/L; thyroid-stimulating antibody, 1078%; thyroid peroxidase antibody, <9.0 U/mL; thyroglobulin antibody, 18.4 U/mL. Ultrasonography revealed increased blood flow in the thyroid gland. Based on these findings, we finally diagnosed her with Basedow disease and thought that various fluid retentions and congestion were caused by hyperthyroidism and Basedow disease.

**Table 1:** Laboratory data observed on admission in this subject.

Variable	Result	Reference range
Peripheral blood		
White blood cells (/μL)	4230	3300–8600
Red blood cells (×10^4^/μL)	397	386–492
Hemoglobin (g/dL)	9.8	11.6–14.8
Platelets (×10^4^/μL)	14.4	15.8–34.8
Blood biochemistry		
Total protein (g/dL)	5.0	6.6–8.1
Albumin (g/dL)	2.9	4.1–5.1
Globulin (g/dL)	2.1	2.2–3.4
Total bilirubin (mg/dL)	0.6	0.4–1.5
AST (U/L)	109	7–23
ALT (U/L)	178	13–30
LDH (U/L)	166	124–222
ALP (U/L)	115	106–322
γ-GTP (U/L)	33	9–32
BUN (mg/dL)	7	8–20
Creatinine (mg/dL)	0.46	0.46–0.79
Cholinesterase (U/L)	201	201–421
Uric acid (mg/dL)	3.9	2.6–5.5
Creatine Kinase (U/L)	17	41–153
Amylase (U/L)	42	44–132
CRP (mg/dL)	0.13	<0.14
BNP (pg/mL)	565.9	0.0–18.4
Plasma glucose (mg/dL)	92	
Total cholesterol (mg/dL)	100	142–248
Iron (μg/dL)	17	40–188
Ferritin (ng/mL)	7	5–160
Thyroid marker		
TSH (μU/mL)	<0.010	0.400–6.000
FT3 (pg/mL)	8.14	2.50–4.20
FT4 (ng/dL)	2.02	0.80–1.60
Electrolytes		
Sodium (mmol/L)	142	138–145
Potassium (mmol/L)	3.4	3.6–4.8
Chloride (mmol/L)	108	101–108
IP (mg/dL)	4.7	2.7–4.6
Calcium (mg/dL)	8.1	8.8–10.1
Magnesium (mg/dL)	1.9	1.9–2.6
Coagulation fibrinolytic system marker		
PT-sec (s)	12.2	9.3–12.5
PT-INR	1.09	0.85–1.13
PT-activity (%)	86.4	80.7–125.2
APTT (s)	31.0	26.9–38.1
Fibrinogen (mg/dL)	189	160–380
D-dimer (μg/mL)	5.2	<1.0
Virus antibody		
HBs antigen	(−)	(−)
HCV Ab	(−)	(−)
HA-IgM Ab	(−)	(−)
CMV IgG Ab	12.1 (+)	<2.0
CMV IgM Ab	0.31 (−)	<0.80
EBV anti-VCA IgG Ab	11.3 (+)	<0.5
EBV anti-VCA IgM Ab	0.0 (−)	<0.5
EBV anti-EBNA IgG Ab	3.8 (+)	<0.5
Chlamydia trachomatis IgA	0.18 (−)	<0.90
Chlamydia trachomatis IgG	0.12 (−)	<0.90
Thyroid antibody		
TSAb (%)	1078	<120
TRAb (U/L)	8.7	<1.0
TPOAb (U/mL)	<9.0	<16.0
TgAb (U/mL)	18.4	<28.0

γ-GTP = γ-glutamyltranspeptidase, Ab = antibody, ALP = alkaline phosphatase, APTT = activated partial thromboplastin time, AST = aspartate aminotransferase, ALT = alanine aminotransferase, BUN = blood urea nitrogen, BNP = brain natriuretic peptide, CRP = C-reactive protein, EBNA =  EBV nuclear antigen, EBV = Epstein-Barr virus, FT3 = free triiodothyronine, FT4 = free thyroxine, HBs = hepatitis B surface, HCV = hepatitis C virus, IgA = immunoglobulin A, IgG = immunoglobulin G, IgM = immunoglobulin M, IP = inorganic phosphorus, LDH = lactate dehydrogenase, PT = prothrombin time, PT-INR = PT-international normalized ratio, TgAb = thyroglobulin antibody, TPOAb = thyroid peroxidase antibody, TRAb = thyroid-stimulating hormone receptor antibody, TSAb = thyroid-stimulating antibody, TSH = thyroid-stimulating hormone, VCA = viral capsid antigen.

First, we administered 4 mg/d of torasemide for the treatment of heart failure and pulmonary hypertension. Her heart failure and pulmonary hypertension immediately improved, although the edematous wall thickening of the gallbladder persisted. Second, we started 15 mg/d of thiamazole and 60 mg/d of metoprolol for Basedow disease, and then tapered thiamazole and metoprolol. About 1 month later, her BNP level and liver dysfunction were improved (BNP, 32.6 pg/mL; aspartate aminotransferase, 26 U/L; alanine transaminase, 24 U/L; alkaline phosphatase, 256 U/L; γ-glutamyl transpeptidase, 14 U/L; lactate dehydrogenase, 138 U/L). In addition, her thyroid hormone levels were normalized (thyroid-stimulating hormone, 0.621 μU/mL; free triiodothyronine, 2.59 pg/mL; free thyroxine, 0.51 ng/dL) with 5 mg/d of thiamazole about 2 months later. At that time, we performed chest and abdominal CT again (Fig. 2, right panel), and her symptoms improved. Chest and abdominal CT revealed the disappearance of edematous wall thickening of the gallbladder, which was likely associated with improvement in thyroid function. Moreover, the periportal collar sign, the increase in inferior vena cava diameter with heart failure, and splenomegaly were improved, and pleural effusion and ascites also disappeared, probably due to the improvement of thyroid hormones.

## 3 Discussion and conclusions

Herein, we report a case of edematous gallbladder wall thickening associated with fluid retention and congestion. In addition, this case is very rare and interesting because her edematous wall thickening of the gallbladder was induced by hyperthyroidism and Basedow disease. Edematous wall thickening of the gallbladder is different from infectious wall thickening of the gallbladder. In general, infectious wall thickening of the gallbladder is associated with infectious diseases, such as cholecystitis; therefore, in many cases, there is gallbladder distention and diffuse wall thickening. In contrast, edematous wall thickening of the gallbladder is caused by congestion of venous blood, which is associated with heart failure, hypoproteinemia, acute hepatitis, and liver cirrhosis. Reflecting congestion of venous blood, the features of edematous wall thickening of the gallbladder on abdominal CT are subserosal edema and periportal collar sign, which likely represent periportal edema. In our patient, edematous wall thickening of the gallbladder was observed on abdominal CT. In addition, since she was complicated by liver dysfunction, we checked for viral infection associated with the liver. In addition, we evaluated thyroid hormones because thyroid disease can result in fluid retention. Hepatitis virus antibodies showed a pattern of prior infection. However, we detected hyperthyroidism and diagnosed the patient with Basedow disease. Therefore, we concluded that the pathology of the edematous wall thickening of the gallbladder was caused by hyperthyroidism and its complications, such as fluid retention and heart failure.

In general, it is well known that nonpitting edema is caused by hypothyroidism. However, hyperthyroidism causes fluid retention with volume overload owing to congestive heart failure. In addition, hyperthyroidism and Basedow disease sometimes cause mild pulmonary hypertension, although severe pulmonary hypertension is rare in patients with Basedow disease.^[6]^ Our patient had mild pulmonary hypertension on echocardiography, and it seemed that she had heart failure due to elevated BNP levels. Her pulmonary hypertension and heart failure were caused by fluid retention and congestion, which seemed to have caused edematous wall thickening of the gallbladder. Moreover, she had liver dysfunction, which was often complicated by Basedow disease. Therefore, we initially considered that the liver and gallbladder were associated with the causes of fluid retention and congestion with heart failure. Her subjective symptoms improved, with improvement in heart failure and pulmonary hypertension. However, her edematous wall thickening of the gallbladder persisted until the hyperthyroidism improved. Therefore, we believe that edematous wall thickening of the gallbladder was at least in part associated with hyperthyroidism and Basedow disease, although the main factors of edematous wall thickening of the gallbladder might have been heart failure and pulmonary hypertension.

Her heart failure may have been caused by other factors. Chronic heart failure is a chronic condition of heart failure or valvular heart disease that occurs mainly in elderly patients. On the other hand, acute heart failure is mainly caused by acute myocardial infarction or excessive stress. She was 30 years old, and her electrocardiogram and echocardiography did not show any possibility of acute myocardial infarction or valvular heart disease. Since she was the mother of 2 children, she might have been under excessive stress. However, since her fluid retention improved after normalization of thyroid function, it was likely that her various fluid retentions and heart failure were mainly caused by hyperthyroidism.

Taken together, we should bear in mind that hyperthyroidism and Basedow disease could be accompanied by edematous wall thickening of the gallbladder and various fluid retentions as the first symptoms associated with heart failure and pulmonary hypertension. In addition, we should know that such edematous wall thickening of the gallbladder and various fluid retentions could be reduced together with the improvement of hyperthyroidism and Basedow disease. Therefore, when examining subjects with edematous wall thickening of the gallbladder, the possibility of hyperthyroidism and Basedow disease should be considered.

## Author contributions

**Conceptualization:** Takatoshi Anno.

**Data curation:** Katsumasa Koyama, Takatoshi Anno, Fumiko Kawasaki, Ken Nishino.

**Formal analysis:** Katsumasa Koyama, Takatoshi Anno, Fumiko Kawasaki, Ken Nishino, Hideaki Kaneto.

**Investigation:** Katsumasa Koyama, Takatoshi Anno, Fumiko Kawasaki, Ken Nishino, Hirofumi Kawamoto.

**Methodology:** Takatoshi Anno.

**Visualization:** Takatoshi Anno.

**Writing – original draft:** Takatoshi Anno, Hideaki Kaneto.

**Writing – review & editing:** Takatoshi Anno, Hirofumi Kawamoto, Hideaki Kaneto, Koichi Tomoda.
